# Construction of a nomogram model for deep vein thrombosis in patients with tibial plateau fracture based on the Systemic Inflammatory Response Index

**DOI:** 10.1186/s12891-024-07328-x

**Published:** 2024-03-27

**Authors:** He Ling, Wencai Li, Zhao Huang, Yonghui Lao, Gaoyong Deng, Rongbin Lu, Wei Su

**Affiliations:** Department Orthopedics Trauma and Hand Surgery, the First Affiliated Hospital of Guangxi Medical University, No. 6 ShuangYong Road, Nanning, Guangxi 530022 China

**Keywords:** Tibial plateau fracture, Deep vein thrombosis, Systemic Inflammatory Response Index, Nomogram

## Abstract

**Background:**

In recent years, the incidence of tibial plateau fracture has been on the rise, predominantly affecting the elderly population. Deep vein thrombosis may lead to poor prognosis in patients. the Systemic Inflammatory Response Index are novel biomarkers of inflammation, and this study aims to verify their predictive effect and construct the nomogram model.

**Method:**

This study used binary logistic regression analysis to predict the predictive effect of SIRI on the occurrence of DVT in tibial plateau fracture patients. And use R studio to construct nomogram model.

**Result:**

The results showed that NC (7.036 [3.516, 14.080], *p* < 0.001), LYM (0.507 [0.265, 0.969], *p* = 0.04), and SIRI (2.090 [1.044, 4.182], *p* = 0.037) were independent predictive factors for DVT. The nomogram demonstrated good predictive performance with small errors in both the training and validation groups, and most clinical patients could benefit from them.

**Conclusion:**

The nomogram constructed based on SIRI can assist clinicians in early assessment of the probability of DVT occurrence.

## Introduction

Tibial Plateau Fracture (TPF) is a common lower limb fracture, accounting for approximately 1% of all lower limb fractures [1]. TPF is an intra-articular fracture typically caused by high-energy trauma, often accompanied by soft tissue and vascular injuries around the knee joint. This can result in joint instability and have a significant impact on lower limb function [2, 3]. There have been numerous studies on the etiology and epidemiological analysis of TPF. Lv et al. [4] conducted an analysis of causes and blood types, finding that high-energy trauma and blood type B increase the likelihood of TPF combined with meniscal injury. Yao et al. [5] performed three-dimensional reconstructions of imaging files from 364 patients with tibial plateau fractures, revealing different fracture line distribution characteristics among various types of TPF, as well as significant differences between different fracture segments. However, there is still limited research on the blood markers associated with tibial plateau fractures. Blood tests, being a common admission examination, often provide important guidance for clinical treatment.

Deep vein thrombosis (DVT) is a common complication in orthopedic surgery, and its incidence has been increasing in recent years. As a serious blood-related complication, once a thrombus detaches from the lower extremities, it can cause pulmonary embolism and lead to severe consequences such as sudden death. Studies have shown that in elderly patients with hip fractures, the incidence of DVT is as high as 20.10% to 31.36% when DVT screening is performed upon admission, and 21.93% of elderly hip fracture patients develop DVT within 24 h after injury [6]. Early-stage DVT often presents with asymptomatic characteristics, and most researchers suggest that early diagnosis of DVT and early intervention and treatment can significantly reduce the occurrence of fatal outcomes [7]. Diagnosing DVT can be assisted by the Wells scoring system [8, 9] and ultrasound examination. The Wells scoring system's characteristics of low specificity and high sensitivity have been well-documented in numerous studies [10, 11]. However, due to the unique demographic situation in China, with a large base of patients, it is often less employed in clinical settings. Currently, the diagnosis of DVT in clinical practice mostly relies on vascular color Doppler ultrasound. However, conditions such as limb swelling due to fractures or open wounds often affect the diagnosis of DVT using color Doppler ultrasound. Some clinical departments may not be able to perform lower limb vascular color Doppler ultrasound examination in a timely manner due to the doctor's experience. Therefore, there is a need for more intuitive, simple, and early predictive indicators for DVT to meet the goal of early prevention and treatment in clinical practice [12].

Numerous studies have indicated that neutrophils, as one of the most important inflammatory factors, are involved in the formation of deep vein thrombosis (DVT). Activated neutrophils can activate endothelial cells, and activated endothelial cells can trigger the release of neutrophil extracellular traps (NETs) [13]. As a specific release product of neutrophils, NETs consist of decondensed chromatin and nuclear proteins, including histones and some granule proteins [14]. NETs can cause endothelial cell toxicity and damage, and neutrophils themselves can also influence certain processes of cell apoptosis, promoting thrombus formation [15]. Currently, many researchers have focused on novel inflammatory factors to achieve better prediction of DVT occurrence [16]. The systemic inflammation response index (SIRI), as a popular inflammatory factor in recent years, has been shown to be a predictive indicator for DVT occurrence after total knee arthroplasty [17]. Muresan et al. [18] retrospectively validated SIRI as an independent risk factor for DVT and constructed a line chart for prediction.

However, currently, there is no research that clearly indicates the relationship between SIRI and the occurrence of deep vein thrombosis (DVT) in patients with tibial plateau fractures. Therefore, this study aims to conduct a retrospective study on tibial plateau fracture patients who were admitted to the hospital from January 2015 to September 2023. The goal is to collect blood routine and biochemical examination results upon admission and construct a predictive model for the occurrence of lower limb deep vein thrombosis in tibial plateau fracture patients based on the Systemic Inflammatory Response Index at admission. This model aims to provide a low-cost, non-invasive, early-stage, and simple prediction tool to assist clinical doctors in making early decisions.

## Materials and methods

### Patient section

This is a single-center retrospective cohort study from China, which collected relevant case data and analyzed it using statistical methods to draw relevant conclusions. The research process and writing will strictly follow the STROBE guidelines. A total of TPF patients diagnosed at the First Affiliated Hospital of Guangxi Medical from January 2015 to September 2023 were collected this study. The inclusion were as follows (Table 1): patients diagnosed tibial plateau fractures diagnostic criteria: (a) a history of knee trauma; (b) clinical manifestations: and limited mobility in the affected knee, ecchymosis the skin, deformity in affected limb, positive longitudinal percussion pain in the lower limb, tenderness and percussion pain at the tibial tuberosity of the affected limb; (c) imaging examination: X-ray and CT indicating discontinuity of the bone cortex of the tibial plateau on the affected side or with significant displacement. The exclusion criteria were as follows (Table 1): (a) inability to obtain results of vascular color Doppler ultrasound; (b) Blood routine test results can’t be obtained; (c) diagnosis of multiple fractures or pathological fractures; (d)recent use of anticoagulants or antiplatelet drugs; (e) concomitant immune system and hematological diseases. This study was approved by the First Affiliated Hospital of Guangxi Medical University Ethics Review Committee (Approval Number: 2023-E583-01). As this is a retrospective study, the ethics committee approved the study without requiring patients to sign informed consent forms, in accordance with Chinese laws and institutional agreements. First Affiliated Hospital of Guangxi Medical University Ethics Review Committee give exemption of informed consent for this study. In this study, patients' personal identifying information will be anonymized.

**Table 1:** Summary Table of inclusion and exclusion criteria

Inclusion Criteria (*n* = 310)	Excluded Criteria (*n* = 60)
(a). A history of knee trauma	(a). Inability to obtain results of vascular color Doppler ultrasound (*n* = 15)
(b). Clinical manifestations: and limited mobility in the affected knee, ecchymosis the skin, deformity in affected limb, positive longitudinal percussion pain in the lower limb, tenderness and percussion pain at the tibial tuberosity of the affected limb	(b). Blood routine test results can’t be obtained. (*n* = 30)
	(c). Diagnosis of multiple fractures or pathological fractures. (*n* = 0)
(c). Imaging examination: X-ray and CT indicating discontinuity of the bone cortex of the tibial plateau on the affected side or with significant displacement	(d). Recent use of anticoagulants or antiplatelet drugs. (*n* = 15)
	(e). Concomitant immune system and hematological diseases. (*n* = 0)

### Data collection and definition

This study collected baseline clinical data and laboratory test, including complete blood count, blood biochemistry, and coagulation function. The baseline clinical data included gender, age, affected side, history of hypertension, history of diabetes, history of alcohol consumption, and smoking history. History of hypertension was defined as a previous diagnosis of hypertension, and history of diabetes was defined as a previous diagnosis of diabetes. Additionally, this study also collected laboratory data upon patient admission, including white blood cell count (WBC), red blood cell count (RBC), hemoglobin (HGB), mean corpuscular hemoglobin concentration (MCHC), mean corpuscular volume (MCV), neutrophils (NC), lymphocytes (LYM), monocytes (MONO), eosinophils (Eos), basophils (Baso), albumin (ALB), aspartate aminotransferase (AST), alanine aminotransferase (ALT), total bilirubin (TBIL), creatinine (Cr), blood urea nitrogen (BUN), prothrombin time (PT), activated partial thromboplastin time (APTT), fibrinogen (FIB), and other relevant data. The inflammatory factor validated in this study is calculated as the systemic inflammatory response index (SIRI) using the formula: SIRI = (neutrophil count × monocyte count)/lymphocyte count [19].

### Outcome

This study considers the occurrence of deep vein thrombosis (DVT) in patients with tibial plateau fractures as the outcome event. The presence of DVT is defined as a positive result, while the absence of DVT is defined as a negative result. Color Doppler ultrasound of the blood vessels is considered the gold standard for diagnosing DVT. Skilled ultrasound physicians perform color Doppler ultrasound examinations of the lower extremity blood vessels and provide diagnostic reports to determine the presence of DVT.

### Statistical analysis

Firstly, this study grouped the data based on the presence or absence of DVT and compared various clinical and laboratory data using SPSS 21.0 (SPSS Inc., Chicago, IL). For continuous data, the Shapiro–Wilk test was used to assess normality. If the data followed a normal distribution, it was presented as mean ± standard deviation, and group comparisons were performed using independent one-way analysis of variance (ANOVA). If the data did not follow a normal distribution, it was presented as median (25th percentile, 75th percentile), and group comparisons were performed using the Kruskal–Wallis test. Categorical data were described using frequencies (percentages), and group comparisons were performed using the chi-square test or Fisher's exact test. Graphpad Prism 9.5.0 was used to create box plots of SIRI in the DVT and non-DVT groups, as well as to plot the ROC curve and calculate the area under the curve. A bilateral *p*-value less than 0.05 was considered statistically significant.

Secondly, the collected independent variables in the data were classified into two groups based on the mean value. Variables below the mean were defined as group 1, while variables above the mean were defined as group 2. SPSS software was used to perform univariate logistic regression analysis on each factor. Subsequently, significant clinical factors from the univariate analysis were included in the multivariate logistic regression analysis to calculate the independent predictive factors for DVT.

Finally, we used SPSS to randomly divide the data into training set and a set in a ratio of 7:3. Based on the of the multivariate logistic regression, we used R Studio (version 4.2.2) to create a nomogram for the independent predictive factors. The predictive performance of the model was evaluated using the ROC curve and the area under the curve (AUC). The model's average error was calculated using a calibration plot, and the clinical utility of the model was analyzed using a decision curve analysis (DCA) plot.

## Results

### Relationship of clinical factors to predictive DVT

**Fig. 1:**
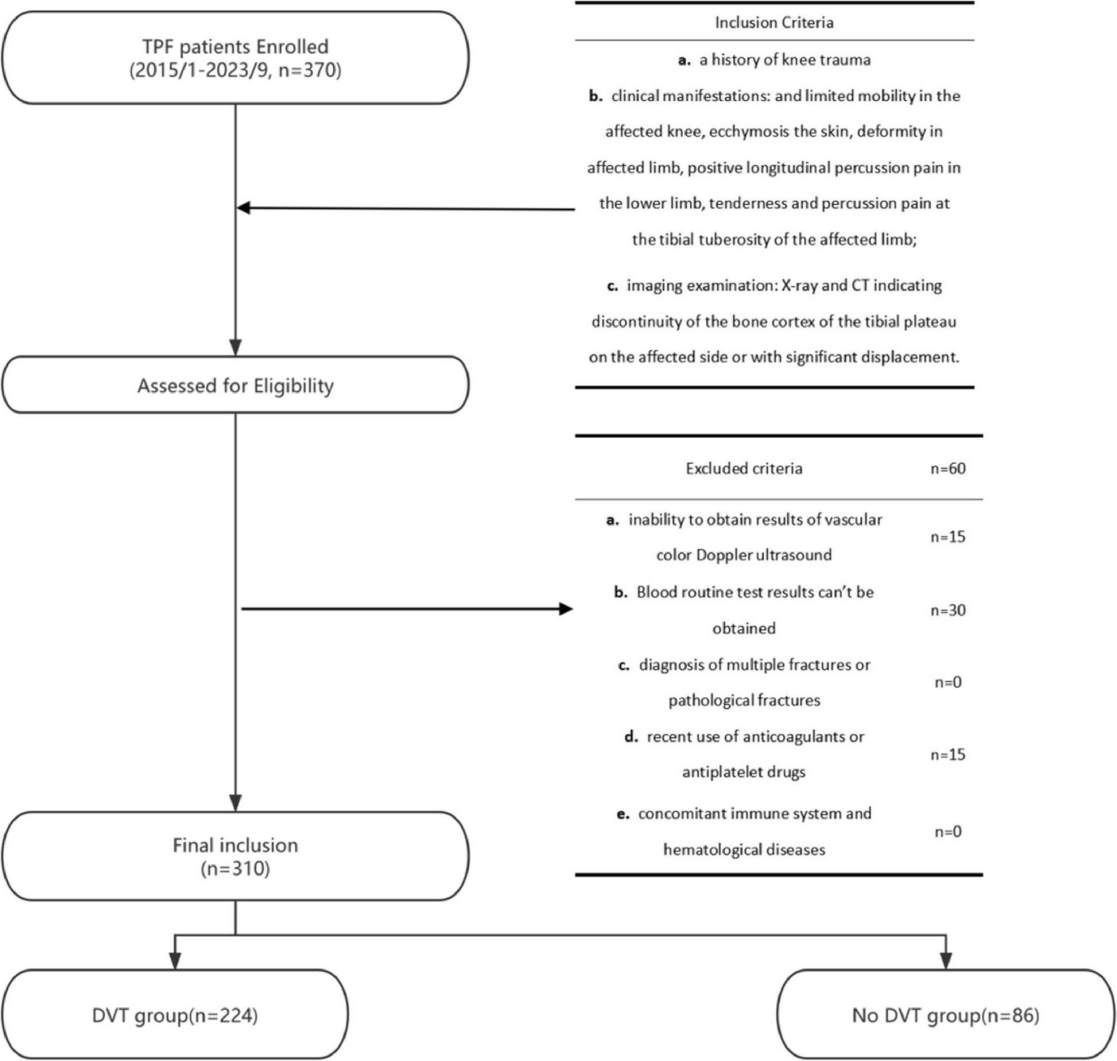
Flow chart of this study. TPF: Tibial plateau fracture

**Fig. 2:**
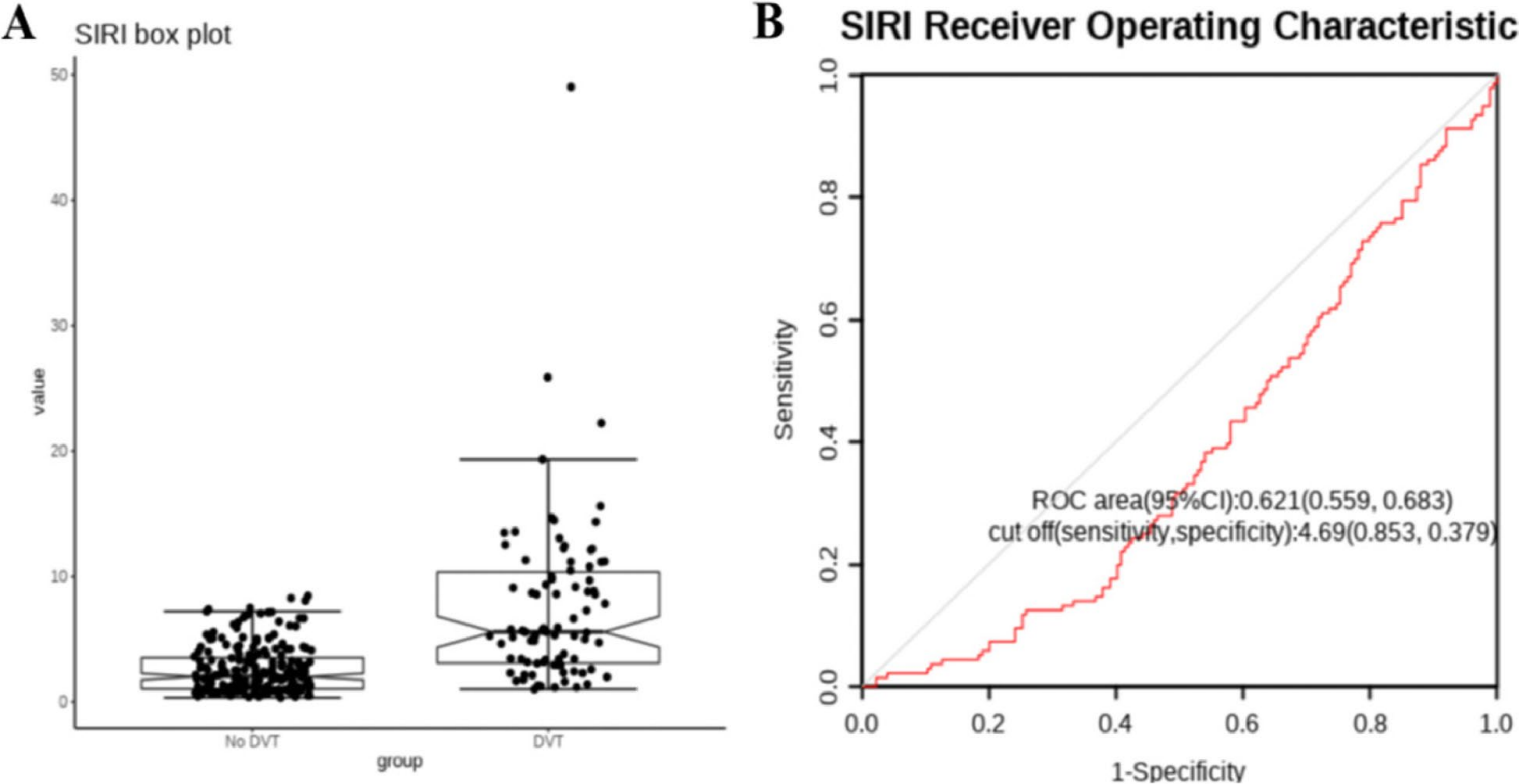
**A** The box plot of SIRI; **B** The ROC of SIRI

A total of 370 patients were included in this study. Among them, 60 patients were excluded due to the aforementioned factors, resulting in a final sample size of 310 patients for the retrospective analysis, as shown in Fig. 1. The data was grouped based on the presence or absence of deep vein thrombosis (DVT). The DVT group, consisting of 224 patients, had a female-to-male ratio of 31:55. The prevalence of comorbidities such as hypertension, diabetes, smoking history, and alcohol abuse in the DVT group was 10.4%, 7.0%, 24.4%, and 8.1% respectively. However, there were no statistically significant differences between the DVT group and the No DVT group in terms of these baseline characteristics (*p* > 0.05). The data was comparable between the two groups. However, there were statistically significant differences between the groups in terms of white blood cell count, absolute neutrophil count, lymphocyte count, monocyte count, systemic immune-inflammation index (SIRI), and albumin levels (*p* < 0.05) (Table 2). Subsequently, a box plot was used to visually display the distribution differences of SIRI between the two groups (Fig. 2A). Additionally, a receiver operating characteristic (ROC) curve was plotted to assess the predictive ability of SIRI for DVT, and the optimal cutoff value, sensitivity, and specificity were calculated (Fig. 2B). The results showed that the optimal cutoff value for SIRI was 0.621, with a sensitivity of 0.853 and a specificity of 0.379.

**Table 2:** Baseline data table for comparison of No DVT and DVT group

Variable	No DVT (*n* = 224)	DVT (*n* = 86)	*p*
Sex			0.085
Male	119(53.1%)	55 (64.0%)	
Female	105(46.9%)	31(36.0%)	
Age	46.75 ± 13.942	50.58 ± 14.077	0.032
Hypertension			0.094
Yes	13(5.8%)	9(10.4%)	
No	199 (88.8%)	65(75.6%)	
Missing	12(5.4%)	12(14.0%)	
Diabetes			0.137
Yes	8(3.6%)	6(7.0%)	
No	204(91.1%)	68(79.0%)	
Missing	12(5.4%)	12(14.0%)	
Smoke			0.945
Yes	59(26.3%)	21(24.4%)	
No	153(68.3%)	53(61.6%)	
Missing	12(5.4%)	12(14.0%)	
Alcoholism			0.649
Yes	25(11.2%)	7(8.1%)	
No	187(83.5%)	67(77.9%)	
Missing	12(5.4%)	12(14.0%)	
BMI	23.58 ± 3.532	23.96 ± 3.151	0.418
RBC	4.36 ± 0.801	4.15 ± 0.758	0.046
HGB	122.77 ± 21.446	118.79 ± 21.181	0.143
MCHC	329.74 ± 11.677	330.49 ± 9.354	0.594
WBC	8.72 ± 2.675	13.64 ± 4.032	< 0.001
NC	6.16 ± 2.592	11.22 ± 3.954	< 0.001
LYM	1.76 ± 0.604	1.42 ± 0.586	< 0.001
MONO	0.62 ± 0.234	0.78 ± 0.394	< 0.001
SIRI	2.56 ± 1.878	7.546 ± 6.726	< 0.001
Eos	0.15 ± 0.176	0.13 ± 0.247	0.366
Baso	0.04 ± 0.028	0.03 ± 0.038	0.271
PCT	0.23 ± 0.080	0.23 ± 0.090	0.346
PT	11.29 ± 1.007	12.10 ± 2.873	0.001
APTT	30.89 ± 3.728	31.09 ± 4.414	0.714
FIB	3.89 ± 1.109	4.01 ± 1.297	0.458
ALB	41.45 ± 4.864	38.84 ± 5.992	< 0.001
GGT	38.22 ± 39.470	38.52 ± 64.485	0.963
ALT	29.55 ± 34.374	40.04 ± 49.250	0.047
AST	33.38 ± 61.220	47.57 ± 82.387	0.120
TBIL	12.21 ± 9.732	13.74 ± 16.401	0.342
Cr	63.88 ± 18.660	70.89 ± 30.218	0.026

The clinical data was dichotomized using the mean value and included in a single-factor binary logistic regression analysis. The results showed that WBC (7.253 [4.106, 12.813]), NC (10.326 [5.680, 18.774]), LYM (0.382 [0.224, 0.651]), MONO (1.961 [1.184, 3.247]), SIRI (6.844 [3.963, 11.820]), Eos (0.559 [0.319, 0.981]), and Baso (0.499 [0.292, 0.852]) were all risk factors for DVT (Table 3). The significant factors identified in the single-factor analysis were then included in a multivariable binary logistic regression analysis. The results showed that NC (7.036 [3.516, 14.080], *p* < 0.001), LYM (0.507 [0.265, 0.969], *p* = 0.04), and SIRI (2.090 [1.044, 4.182], *p* = 0.037) were independent predictive factors for DVT (Table 4).

**Table 3:** Single binary logistic regression analysis results

Variable	OR[95%CI]	*p*
Sex		0.087
Male	1	
Female	0.639[0.383–1.066]	
Age		0.033
≤ 47.8	1	
> 47.8	1.020[1.002–1.038]	
Hypertension		0.100
No	1	
Yes	2.120[0.866–5.186]	
Diabetes		0.146
No	1	
Yes	2.250[0.754–6.716]	
Smoke		0.945
No	1	
Yes	1.021[0.567–1.838]	
Alcoholism		0.649
No	1	
Yes	0.814[0.335–1.976]	
RBC		0.755
≤ 4.3	1	
> 4.3	0.858[0.329–2.240]	
WBC		< 0.001
≤ 10.1	1	
> 10.1	7.253[4.106–12.813]	
HGB		0.704
≤ 121.6	1	
> 121.6	0.908[0.552–1.494]	
NC		< 0.001
≤ 7.6	1	
> 7.6	10.326[5.680–18.774]	
LYM		< 0.001
≤ 1.7	1	
> 1.7	0.382[0.224–0.651]	
MONO		0.009
≤ 0.7	1	
> 0.7	1.961[1.184–3.247]	
SIRI		< 0.001
≤ 3.95	1	
> 3.95	6.844[3.963–11.820]	
Eos		0.043
≤ 0.15	1	
> 0.15	0.559[0.319–0.981]	
Baso		0.011
≤ 0.04	1	
> 0.04	0.499[0.292–0.852]	
PCT		0.348
≤ 0.23	1	
> 0.23	1.271[0.770–2.097]	
PT		0.946
≤ 11.51	1	
> 11.51	1.059[0.201–5.584]	
APTT		0.873
≤ 30.94	1	
> 30.94	0.957[0.558–1.642]	
FIB		0.976
≤ 3.92	1	
> 3.92	0.992[0.576–1.706]	
ALB		0.124
≤ 40.72	1	
> 40.72	0.660[0.389–1.121]	
GGT		0.536
≤ 74.52	1	
> 74.52	1.199[0.675–2.131]	
AST		0.119
> 37.35	1	
≤ 37.35	1.632[0.881–3.021]	
ALT		0.393
> 32.48	1	
≤ 32.48	1.282[0.725–2.268]	
TBIL		0.425
≤ 12.64	1	
> 12.64	1.248[0.724–2.151]	
Cr		0.502
> 65.87	1	
≤ 65.87	1.206[0.698–2.084]

**Table 4:** Multivariate binary logistic regression analysis results

Variable	OR[95%CI]	*p*
NC		< 0.001
≤ 7.6	1	
> 7.6	7.036[3.516–14.080]	
LYM		0.040
≤ 1.7	1	
> 1.7	0.507[0.265–0.969]	
SIRI		0.037
≤ 3.95	1	
> 3.95	2.090[1.044–4.182]	

### Construction and evaluation of nomogram

**Fig. 3:**
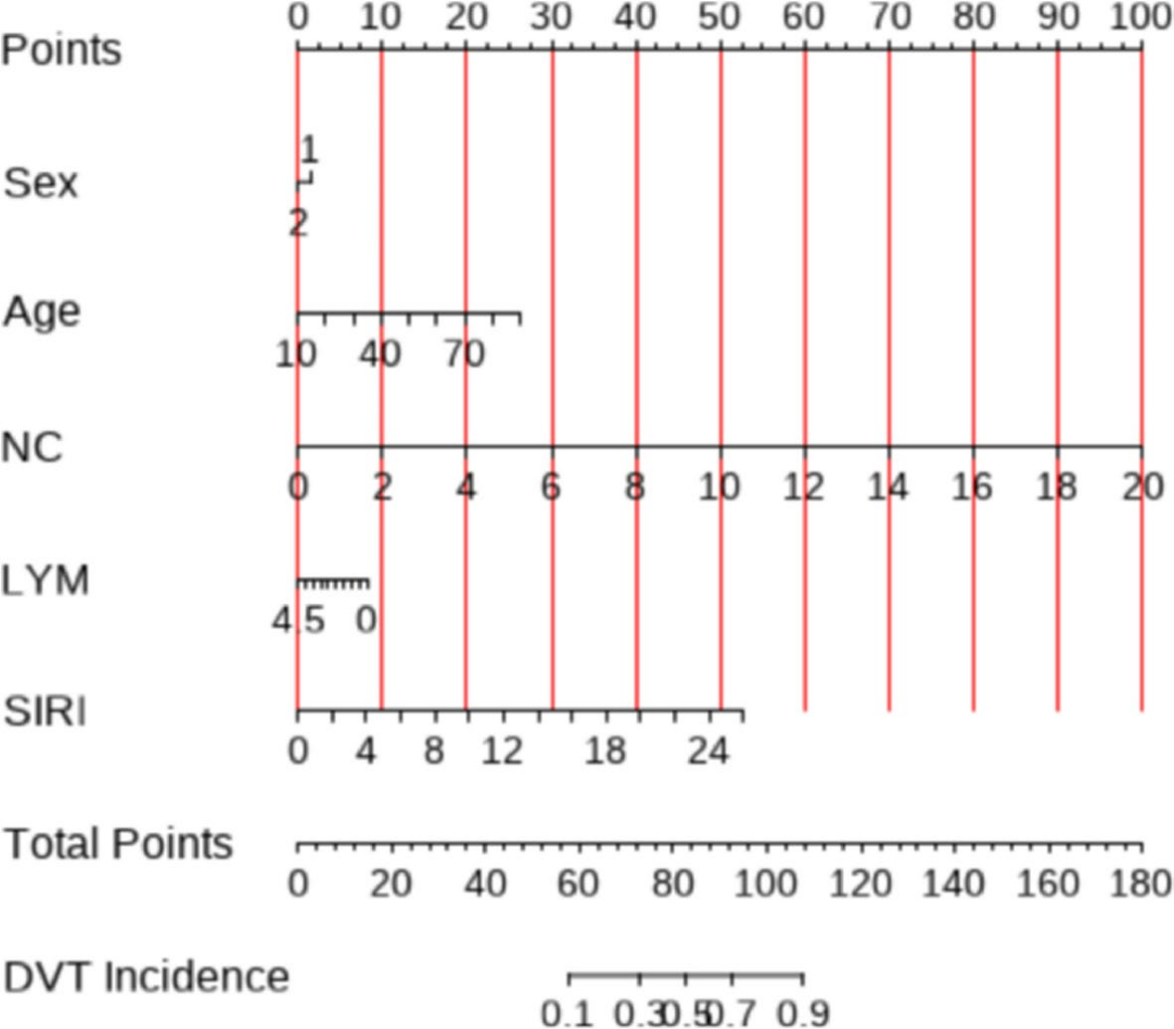
Nomogram of clinical data

**Fig. 4:**
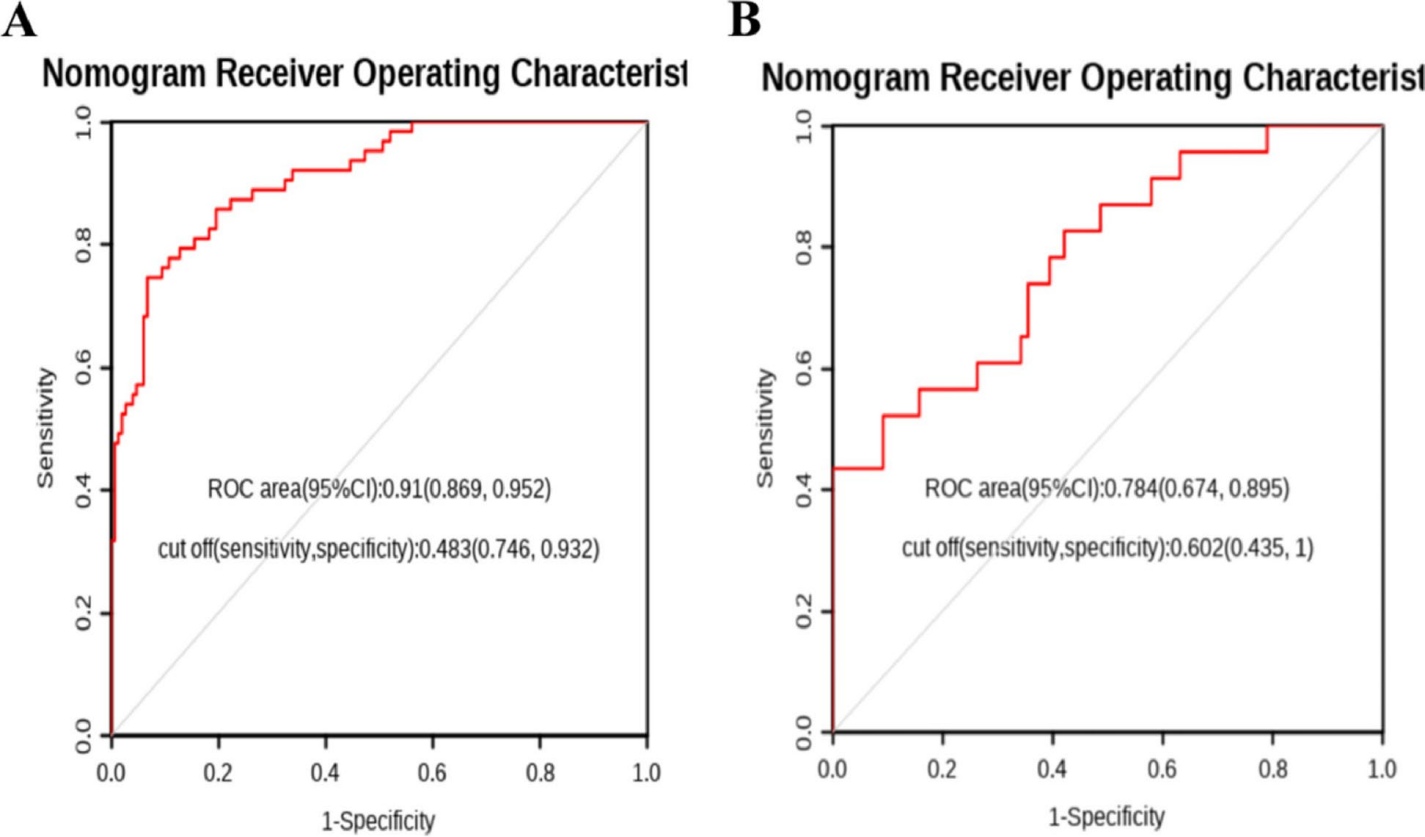
**A** ROC curve of training set data; **B** ROC curve of validation set data

**Fig. 5:**
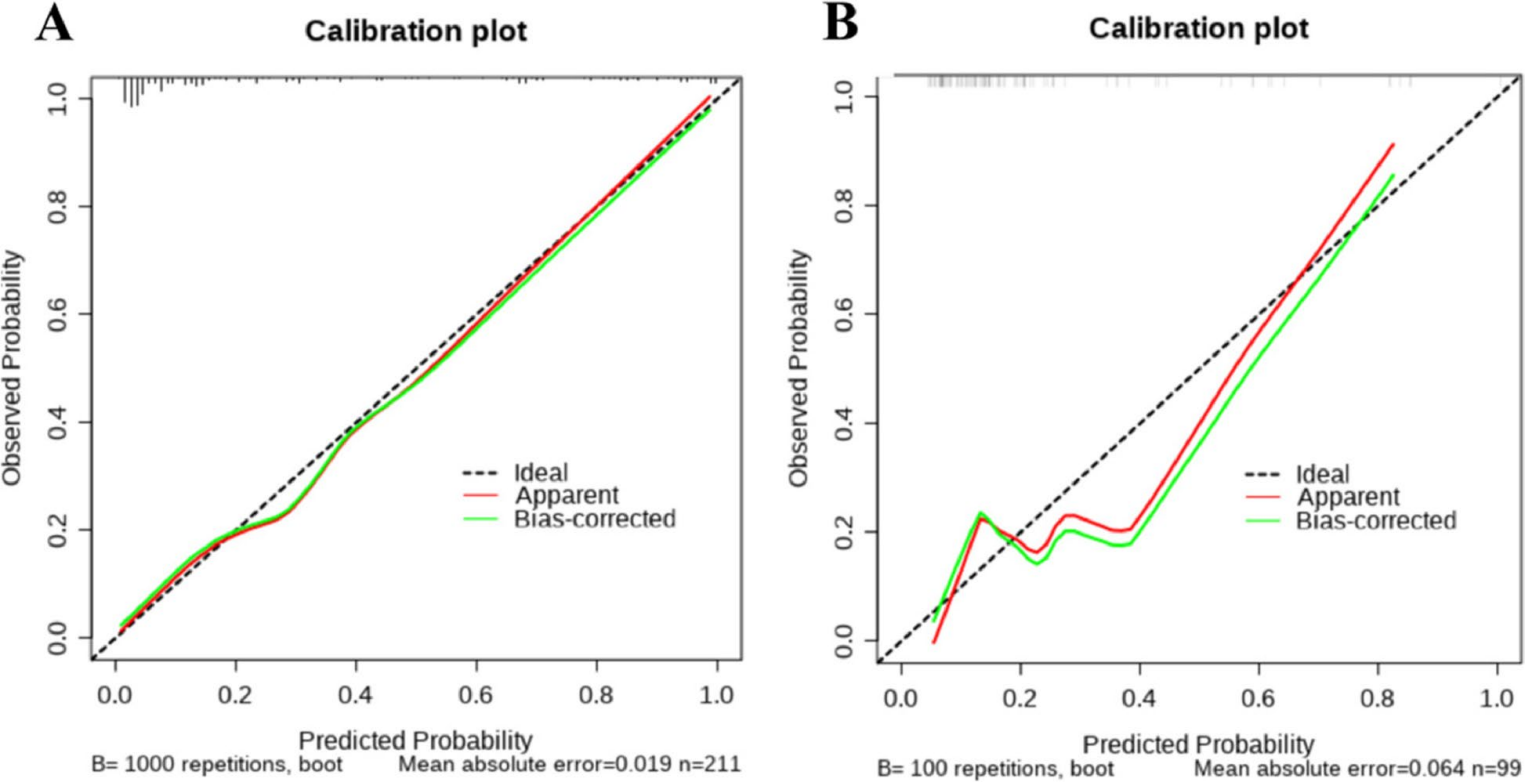
**A** Calibration curve of training set data; **B** Calibration curve of validation set data

**Fig. 6:**
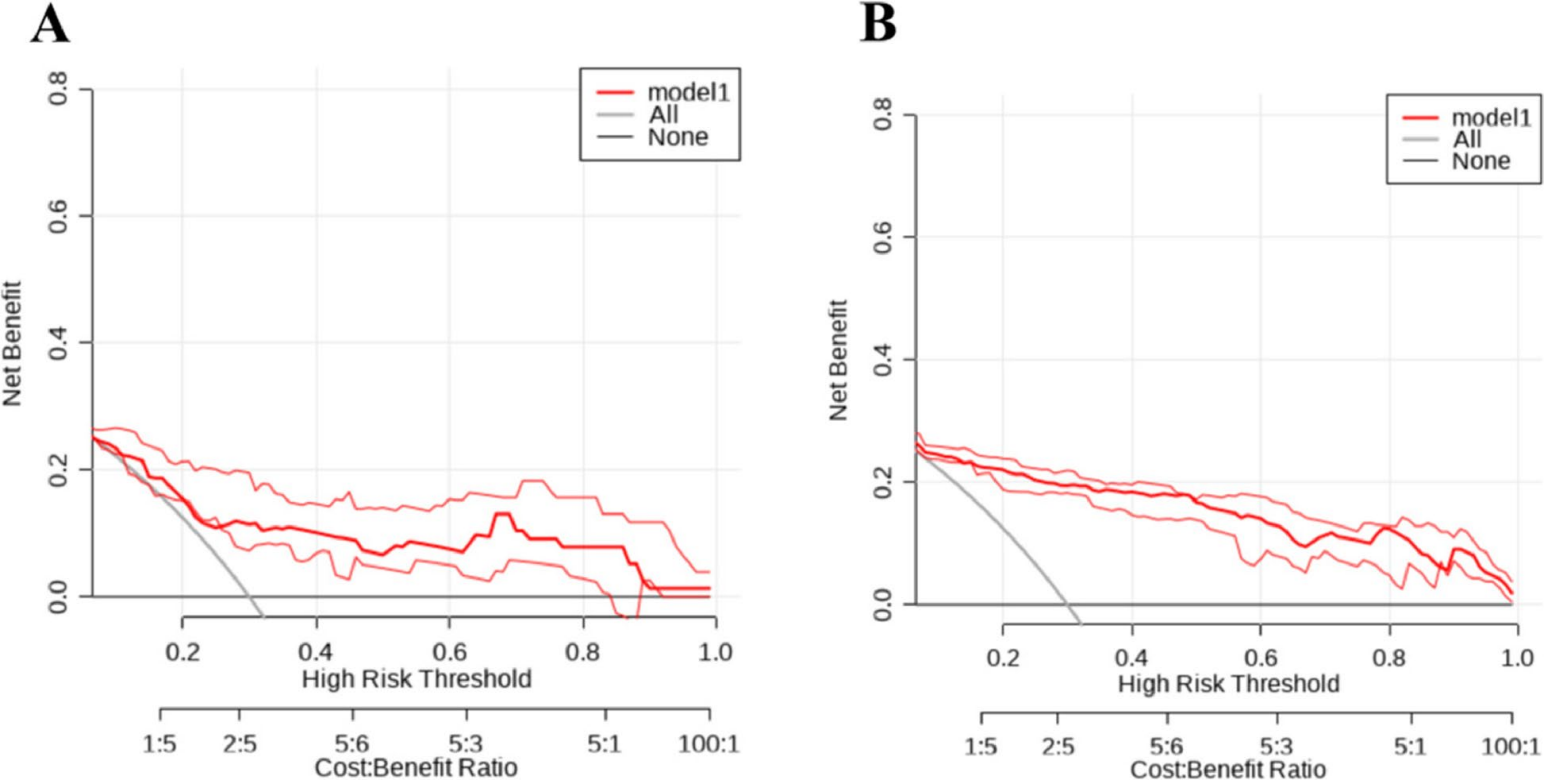
**A** DCA curve of training set data; **B** DCA curve of validation set data

To further validate the predictive ability of the factors for tibial plateau fractures, a nomogram model was constructed based on the results of the multivariable analysis (NC, LYM, SIRI) using R Studio software. The collected data was randomly sampled in a 7:3 ratio and divided into training and validation groups. The training group data was used to construct the nomogram, as shown in Fig. 3. The training group ROC curve was plotted to validate the predictive performance of the nomogram (Fig. 4A), with a C-index of 0.91, indicating good predictive ability. The training group calibration curve (Fig. 5A) showed an average error of 0.019. The training group decision curve analysis (DCA) (Fig. 6A) indicated good clinical benefit within the threshold range of 0.01–0.99. The validation group data was used for further validation. The ROC curve for the validation group (Fig. 4B) had a C-index of 0.784. The calibration curve (Fig. 5B) showed an average error of 0.064. The validation group DCA (Fig. 6B) indicated good clinical benefit within the threshold range of 0.01–0.99. It is evident that the column chart exhibited good predictive performance with small errors in both the training and validation groups. Moreover, it provided clinical benefit to a majority of the patients.

## Discussion

Tibial plateau fractures are common and severe injuries that occur after high-impact trauma. They can occur not only in young patients but also in elderly patients with osteoporosis [20]. Deep vein thrombosis (DVT) is a common complication during the perioperative and postoperative periods, especially in elderly patients, and it often has an impact on patient prognosis. Studies have indicated that impaired venous return in the lower limbs can lead to swelling, discomfort, and gait disturbances. If left untreated, it may progress to pulmonary embolism and death [21]. In hip fractures, the incidence of DVT can reach 46%-75% if preventive measures are not taken, highlighting the necessity of early diagnosis and treatment of DVT [22]. Research has also shown that the occurrence of deep venous thrombosis may be related to inflammatory factors, in addition to hemodynamic influences [23].

Many studies have investigated the mechanisms underlying the association between DVT and inflammatory responses. Trauma can induce vascular inflammation, where an inflammatory cascade occurs in the venous vessels. Monocytes and macrophages aggregate at the site of inflammation and release inflammatory cytokines, leading to acute vascular inflammation. This damages the endothelial cells of the blood vessels and promotes thrombus formation [24].

Currently, many scholars have proposed a close association between the immune system and the formation of DVT. Early studies have indicated that post-traumatic inflammatory responses activate inflammatory cells and release inflammatory cytokines [25]. Neutrophils play a crucial role in thrombus formation, and early trauma stress leads to the activation of neutrophils, exacerbating endothelial tissue damage and accelerating thrombus formation [26]. The inflammatory response in DVT is associated with neutrophil extracellular traps, which activate and initiate the endogenous coagulation pathway. Simultaneously, monocytes trigger the exogenous coagulation pathway, thereby promoting venous thrombus formation [27, 28]. Therefore, a procoagulant state is induced by a series of proinflammatory factors, and the inflammatory process increases the risk of DVT [29]. In recent years, there has been an increasing amount of research on the predictive role of inflammatory factors in DVT events. Systemic immune-inflammation index (SIRI), as a novel inflammatory marker reflecting the inflammatory status, has been widely used in various diseases [30–32]. In this study, through logistic regression analysis and nomogram model construction, the predictive role of SIRI was fully validated, enabling early prediction of DVT occurrence in the very early stages and facilitating early preventive treatment. Based on the SIRI inflammatory biomarkers, the construction of the nomogram in this study demonstrated good predictive performance with small average errors, providing significant clinical benefits. It can assist clinicians in making relevant predictions and judgments upon patient admission, thereby implementing appropriate preventive measures.

This study has several strengths: (1) It is a retrospective study that constructs a model using a training set and validates it with a validation set, making the model reliable and easy to apply. (2) Routine blood tests and biochemical examinations are usually conducted upon patient admission. This study aims to collect relevant data at the time of admission to achieve early prediction and benefit more patients. However, there are also some limitations to this study: (1) The postoperative and long-term incidence rates of patients were not tracked. (2) The study could further expand the number of centers and data volume.

## Conclusion

The admission levels of NC, LYM, and SIRI were identified as independent predictors of deep vein thrombosis (DVT) in patients with tibial plateau fractures. The construction of a bar chart based on SIRI can assist clinicians in early assessment of the probability of DVT occurrence upon patient admission, thereby achieving the goal of early prevention and treatment and benefiting a larger number of patients. This early assessment and intervention can reduce the risk of DVT and improve patient recovery and quality of life.

## Data Availability

The data used to support the findings of this study are available from the corresponding author upon request.

## Abbreviations

DVT: Deep venous thrombosis
TPF: Tibial plateau fracture
WBC: White blood cell count
RBC: Red blood cell count
HGB: Hemoglobin
MCV: Mean corpuscular volume
MCHC: Mean corpuscular hemoglobin concentration
NC: Neutrophils
LYM: Lymphocyte
MONO: Monocyte
Eos: Eosinophils
Baso: Basophil
ALB: Albumin
AST: Aspartate aminotransferase
ALT: Alanine aminotransferase
TBIL: Total bilirubin
PT: Prothrombin time
APTT: Activated partial thrombin time
FIB: Fibrinogen
Cr: Creatinine
BUN: Blood urea nitrogen
SIRI: Systemic inflammatory response index
OR: Odds ratio
CI: Confidence interval
AUC: The area under the curve
DCA: Decision curve analysis
ROC: Receiver operating characteristic
